# Hepatitis B surface antigen is upregulated by HIV Tat in an HIV–hepatitis B virus co-infection model system

**DOI:** 10.1128/spectrum.00809-25

**Published:** 2025-07-23

**Authors:** Wei Zhao, Kasha Singh, Vitini Sozzi, Paula Cevaal, Fernando J. Rossello, Luciano Martelotto, Jennifer M. Zerbato, Ajantha Rhodes, David R. Powell, Jan Schröder, Jenny Anderson, Carolin Tumpach, Danielle Fong, Peter Revill, Megan Crane, Sean Grimmond, Michael Roche, Jennifer Audsley, Sharon R. Lewin

**Affiliations:** 1 Department of Infectious Diseases, The University of Melbourne at the Peter Doherty Institute for Infection and Immunity, Melbourne, Victoria, Australia; 2 Victorian Infectious Diseases Service, Royal Melbourne Hospital at the Peter Doherty Institute for Infection and Immunity, Melbourne, Victoria, Australia; 3 Department of Infectious Diseases, Alfred Health and Monash University, Melbourne, Victoria, Australia; 4 Victorian Infectious Diseases Reference Laboratory, Royal Melbourne Hospital at the Peter Doherty Institute of Infection and Immunity, Melbourne, Victoria, Australia; 5 Murdoch Children’s Research Institute, The Royal Children’s Hospital, Melbourne, Victoria, Australia; 6 Novo Nordisk Foundation Center for Stem Cell Medicine, Murdoch Children’s Research Institute, Melbourne, Victoria, Australia; 7 Department of Clinical Pathology, University of Melbourne 198085, Melbourne, Victoria, Australia; 8 Australian Regenerative Medicine Institute, Monash University 2541, Melbourne, Victoria, Australia; 9 Adelaide Centre for Epigenetics, South Australian Immunogenomics Cancer Institute 827032 , Adelaide, South Australia, Australia; 10 Monash Bioinformatics Platform, Monash University 2541, Melbourne, Australia; 11 Computational Sciences Initiative, Department of Microbiology and Immunology, Peter Doherty Institute for Infection and Immunity, University of Melbourne 2281, Melbourne, Victoria, Australia; 12 The University of Melbourne Centre for Cancer Research, The University of Melbourne 2281, Melbourne, Victoria, Australia; David Geffen School of Medicine at UCLA, Los Angeles, California, USA

**Keywords:** human immunodeficiency virus, hepatitis B virus, co-infection, Tat, liver, CDK9, HBsAg, hepatocyte

## Abstract

People with human immunodeficiency virus-hepatitis B virus (HIV-HBV) co-infection have faster rates of liver disease progression and an increase in hepatocellular carcinoma compared to people with HBV mono-infection. Given that HIV can infect multiple cells in the liver, including hepatocytes, we hypothesized that HIV will impact HBV replication through HIV viral proteins that can impact HBV replication either directly or indirectly, via effects on cellular pathways. Following infection of sodium taurocholate co-transporting polypeptide (NTCP)-expressing HepG2 cells with HBV and vesicular stomatitis virus G protein (VSV.G)-pseudotyped HIV, we found that productive HIV infection led to a twofold upregulation of HBV surface (HBs) mRNA and a marked increase in intracellular production and cellular retention of HBs antigen (HBsAg). Overexpression of HIV Tat protein, but not other HIV proteins, by DNA plasmid transfection in the HBV-producing cell line AD38 significantly stimulated HBs mRNA expression. This could be rescued by CDK9 inhibition with BAY-1251152. This study provides new insights into the mechanisms by which HIV directly impacts HBV replication and has implications for understanding adverse liver outcomes in people living with HIV and HBV.

**IMPORTANCE:**

People with both human immunodeficiency virus (HIV) and hepatitis B virus (HBV) face faster liver disease progression and a higher risk of liver cancer than those with HBV alone. This study investigated how HIV affects HBV replication in liver cells and found that HIV infection increases the production of a key HBV surface protein (HBsAg) by enhancing the expression of its gene (HBs). This effect is driven by the HIV Tat protein. Notably, blocking the CDK9 pathway prevented this increase, suggesting a possible explanation for the adverse liver outcomes in co-infected individuals. Our findings have implications for interventions aiming to cure HIV through latency reversal, as these interventions can specifically increase the Tat protein. Future exploratory treatment strategies, such as Tat inhibitors, could play a role in the management of people with HIV and HBV at high risk of liver disease.

## INTRODUCTION

Approximately 7.4% of people living with HIV (PLWH) are co-infected with hepatitis B virus (HBV), meaning that about 2.7 million people are living with both viruses worldwide (1). Current treatment with HBV-active antiretroviral therapy (ART) including antivirals, such as tenofovir (TDF), tenofovir alafenamide (TAF), lamivudine (LMV), or emtricitabine (FTC), can fully suppress both HIV and HBV replication leading to a significant reduction in liver-related mortality (2, 3). However, overall mortality, liver-related mortality, hospital utilization rates, and the risk of hepatocellular carcinoma (HCC) remain significantly higher in people co-infected with HIV and HBV on HBV-active ART compared to those with HIV or HBV mono-infection (4, 5). Furthermore, we and others have shown that there is liver disease progression in 10–20% of people living with HIV-HBV co-infection despite HBV-active ART (6–9). Understanding how HIV and HBV interact within hepatocytes could potentially identify novel approaches to reduce adverse liver outcomes.

HBV infects hepatocytes leading to the establishment of an episomal covalently closed circular (ccc) DNA mini-chromosome, which serves as a very stable template for HBV RNA transcription (10). Production of RNA includes pregenomic (pg) RNA, which undergoes reverse transcription resulting in HBV DNA that gets packaged into new virions; and pre-S1 and pre-S2 RNA, which are translated to form HBV surface antigen (HBsAg). Antiviral nucleos(t)ide reverse transcriptase inhibitors block the production of HBV DNA but have minimal impact on the ongoing production of HBsAg (11, 12). HBsAg can also be produced from mRNA derived from integrated HBV DNA, which is the dominant source of HBsAg in people with hepatitis B e antigen negative chronic HBV (13). HBsAg can inhibit adaptive immunity and the effective production of anti-HBs antibodies, which are required for long-term HBV control (11). If synthesized in large quantities, HBsAg can also cause direct damage to the hepatocyte through activation of Fas ligand-mediated apoptosis (14). Persistent high levels of HBsAg have also been shown to increase the risk of HCC in untreated HBV mono-infection (15, 16). Therefore, increased production of HBsAg can have a significant impact on a range of clinical outcomes in people living with HBV.

In addition to CD4^+^ T cells, HIV can also infect multiple cells in the liver, potentially impacting liver disease outcomes. We and others have shown that HIV can infect cells in the liver, using *in vitro*, *ex vivo,* and *in vivo* models, including HIV infection of hepatocytes (17, 18), Kupffer cells (19–22), hepatic stellate cells (HSC) (23), and infiltrating T-cells (24). Using a cell line model of immortalized hepatocytes producing HBV, we previously demonstrated that HIV could infect these cells via either CCR5 or CXCR4, despite very low expression of both coreceptors, leading to an increase in intracellular HBsAg (25). Whether the increase in intracellular HBsAg was due to increased production of HBsAg or reduced release remains unknown, as does the mechanism by which HIV impacts levels of intracellular HBsAg.

## RESULTS

### Pseudotyped HIV infection of HBV-expressing hepatocytes leads to efficient HIV integration and production

**Fig 1:**
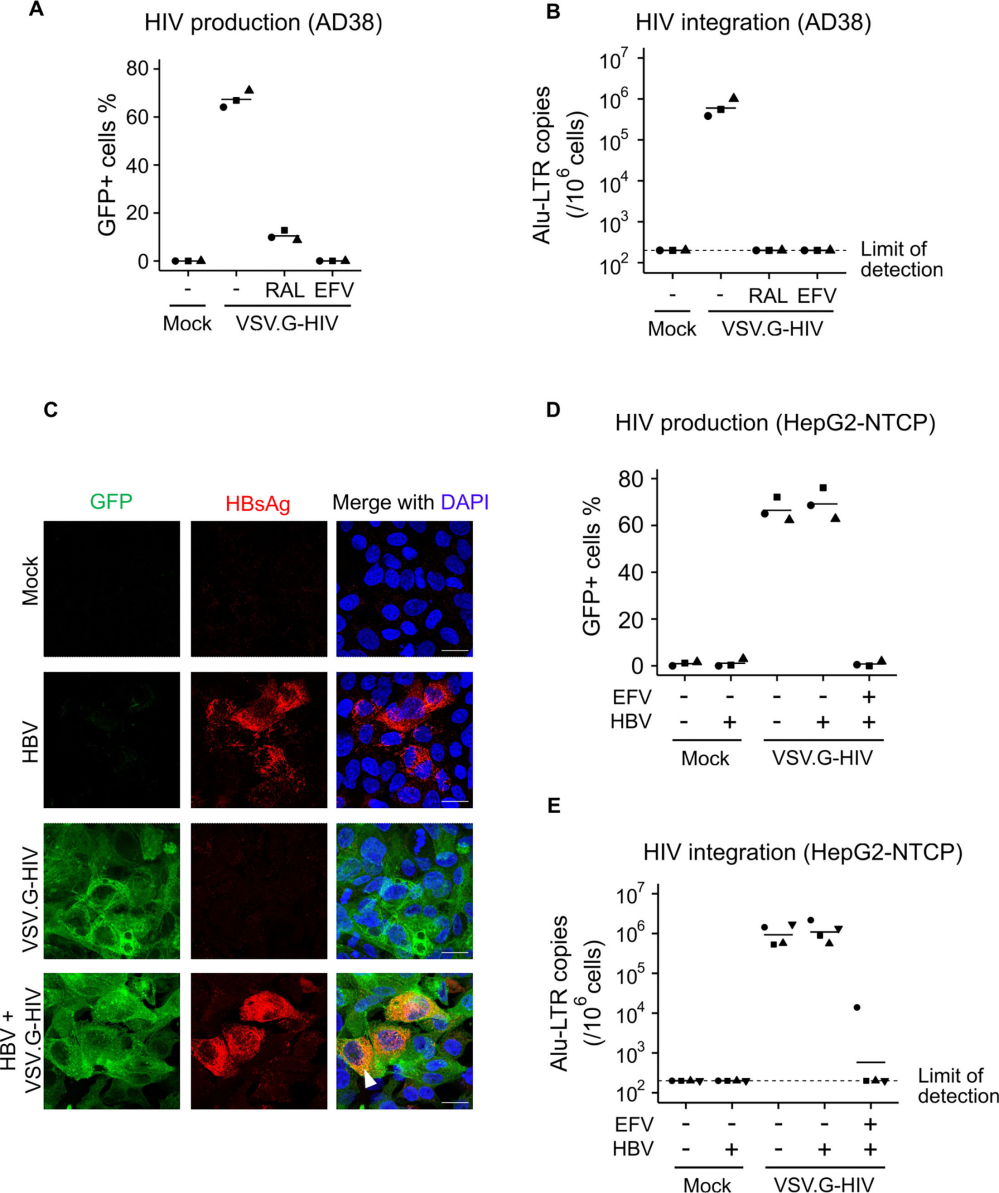
High levels of HIV-HBV co-infection in AD38 and HepG2-NTCP cells using VSV.G pseudotyped HIV virus. (**A**) Percentage of AD38 cells expressing green fluorescent protein (GFP) 5 days post VSV.G-pseudotyped HIV infection. Cells were treated with or without 10 µM raltegravir (RAL) or 300 nM efavirenz (EFV) 24 h before and immediately after HIV infection. GFP expression was determined by flow cytometry (*N* = 3). (**B**) Frequency of HIV integration in AD38 cells 5 days post VSV.G-pseudotyped HIV infection with or without RAL/EFV treatment. HIV integration was measured by real-time PCR for Alu-LTR and normalized to CCR5 copy number. The detection limit for the Alu-LTR was 200 copies/10^6^ cells and is shown as a dashed line (*N* = 3). (**C**) GFP and HBsAg expression in mock, HBV-mono, HIV-mono, and HIV-HBV co-infected HepG2-NTCP cells. HepG2-NTCP cells were infected with HBV inoculum derived from AD38 cells for 10 days and VSV.G-pseudotyped HIV virus for another 5 days. Cells were immunostained for GFP (green) and HBsAg (red). DNA was counterstained with DAPI (blue). Bars, 10 µm. Results are representative of at least three experiments. (**D**) Percentage of HepG2-NTCP cells expressing GFP following HBV and VSV.G-pseudotyped HIV infection. Cells were treated with or without 300 nM EFV 24 h before and after HIV infection (*N* = 3). (**E**) HIV integration in HepG2-NTCP cells following HBV and VSV.G-pseudotyped HIV infection with or without EFV treatment (*N* = 4). In all graphs, the horizontal bar represents the mean.

Direct interaction between HIV and HBV can result in significant adverse outcomes in co-infected cells, even though wild-type HIV only infects hepatic cell lines at very low levels (17, 25). To study these rare but potentially impactful events, we used a vesicular stomatitis virus G protein (VSV.G) pseudotyped, HIV envelope deleted virus that expressed green fluorescent protein (GFP) (VSV.G-NL4-3-ΔEnv-EGFP). This system ensures high and consistent levels of single-round HIV infection. AD38 cells, an immortalized HBV-producing hepatocyte cell line, were first infected with the pseudotyped HIV virus at a multiplicity of infection (MOI) of 0.5 for 4 days. A mean (range) of 67.3% (64.1–71.0%; *N* = 3) cells expressed GFP (Fig. 1A and S1A), with a mean frequency of 650,228 (384,091–1,011,736; *N* = 3) copies of integrated HIV DNA per million cells (Fig. 1B). Addition of the HIV integrase inhibitor raltegravir or the non-nucleoside reverse transcriptase inhibitor efavirenz effectively eliminated GFP expression and detection of HIV integration (Fig. 1A and B and Fig. S1A).

To mimic HBV infection *in vivo*, the sodium taurocholate co-transporting polypeptide (NTCP) expressing cell line, HepG2-NTCP, was next infected with HBV virions concentrated from the supernatant of AD38. We inoculated 800 viral genome equivalents (VGE) per cell for 10 days in the presence or absence of single-round HIV infection using an MOI of 1. GFP expression was detected using fluorescent microscopy with a mean (range) of 69.8% (62.3–72.1%; *N* = 3) of the cells infected with HIV. Staining of intracellular HBsAg confirmed HBV production in a mean (range) of 22.5% (16.7–28.9%; *N* = 3) of the HBV mono-infected cells (Fig. 1C). In cells infected with both HIV and HBV, the expression of HBsAg and GFP was detected in 22.6% (12.8–37.0%; *N* = 4) and 67.6% (62.8–76.1%; *N* = 4) of cells, respectively. 15.5% (9.0–26.0%; *N* = 3) of the cells expressed both HBsAg and GFP (arrowhead in Fig. 1C). Infection with HIV resulted in high levels of GFP expression and HIV integration in the presence or absence of HBV infection, and as expected, HIV infection was inhibited in the presence of efavirenz (Fig. 1D and E). These results demonstrated that HIV-HBV co-infection could be established in the HepG2-NTCP cell line.

### Productive HIV infection induces a significant increase in intracellular HBsAg

**Fig 2:**
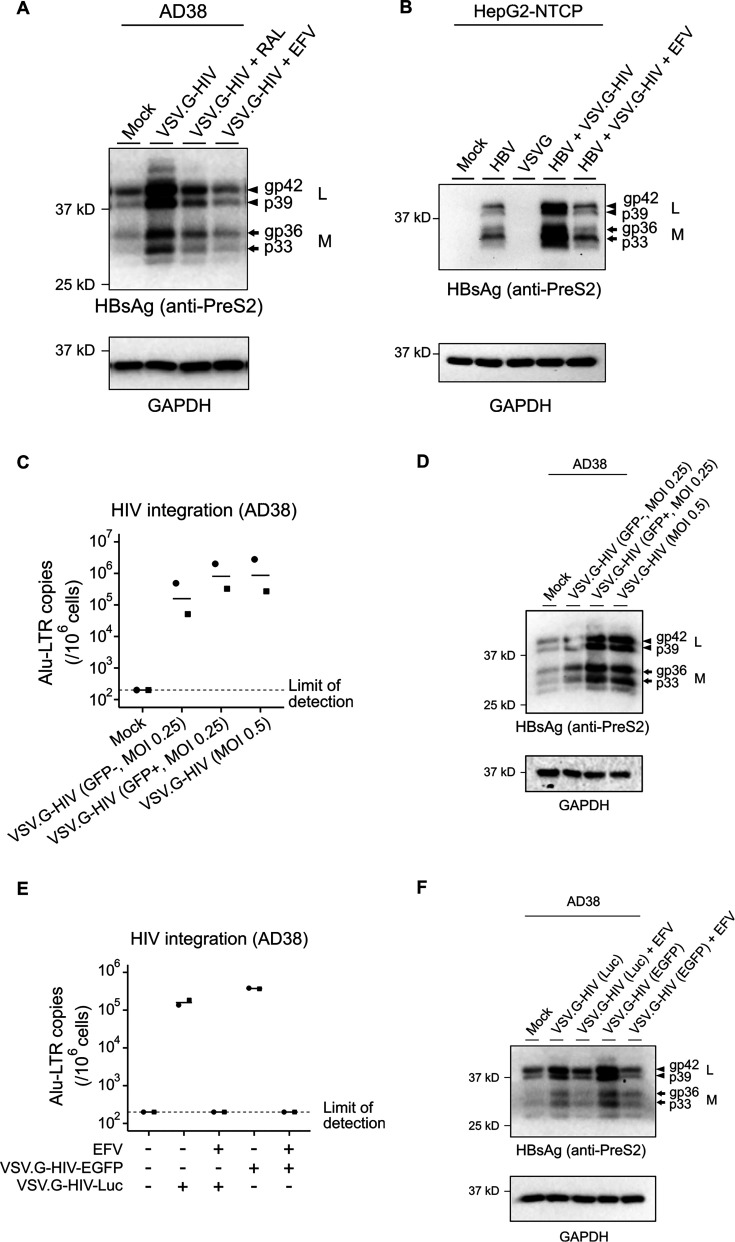
Productive HIV infection leads to an increase in intracellular HBsAg in AD38 and HepG2-NTCP cells. (**A**) Western blot with anti-HBV PreS2 and anti-GAPDH antibodies using lysates from AD38 cells 5 days post-infection with VSV.G-pseudotyped HIV treated with or without 10 µM RAL or 300 nM EFV 24 h before and after HIV infection. Results are representative of at least three experiments. (**B**) Western blot with anti-HBV PreS2 and anti-GAPDH antibodies using lysates from HepG2-NTCP cells infected with HBV, VSV.G-pseudotyped HIV infection or both and treated with or without 300 nM EFV 24 h before and immediately after HIV infection. Results are representative of at least three experiments. Results are representative of at least three experiments. (**C**) HIV integration in AD38 cells with or without productive HIV infection. GFP+ and GFP− cells were collected by flow sorting 5 days post VSV.G-pseudotyped HIV infection. HIV integration was quantified using real-time PCR for Alu-LTR and normalized to CCR5 copy numbers as a housekeeping gene. The detection limit for the Alu-LTR was 200 copies/10^6^ cells and is shown as a dashed line (*N* = 2). (**D**) Representative example (from two separate experiments) of intracellular HBsAg levels in AD38 cells with or without productive HIV infection. GFP+ and GFP− cells were collected by flow sorting 5 days post VSV.G-pseudotyped HIV infection. Cell lysates of unsorted and sorted samples were examined by Western blot with anti-HBV PreS2 and anti-GAPDH antibodies. (**E**) HIV integration in AD38 cells infected with an HIV virus expressing GFP or a luciferase (luc) reporter. Cells were treated with or without 300 nM EFV 24 h before and immediately after HIV infection (*N* = 2). (**F**) Representative example (from two independent experiments) of intracellular HBsAg levels in AD38 cells 5 days post VSV.G-pseudotyped HIV infection expressing GFP or a luciferase (luc) reporter. Cells were treated with or without 300 nM EFV 24 h before and immediately after HIV infection. AD38 cell lysates were examined by Western blot with anti-HBV PreS2 and anti-GAPDH antibodies. In all graphs, the horizontal bar represents the mean.

We then assessed the impact of HIV co-infection on HBV protein production in both AD38 and HepG2-NTCP cell lines. Following HIV infection of AD38 cells (as described above), we detected a substantial increase in intracellular HBsAg (L and M forms) as detected by Western blot using an anti-preS2 antibody (Fig. 2A). This aligns with our previous study showing increases in all HBsAg, including the S form, in the context of HIV co-infection (25). Similar findings were observed following HIV-HBV co-infection of HepG2-NTCP cells (Fig. 2B). Antiretroviral drugs inhibited the increase in HBsAg in both AD38 (Fig. 2A) and HepG2-NTCP cells (Fig. 2B). There was no significant increase in HBsAg in the supernatant following HIV infection of either HBV-infected AD38 or HepG2-NTCP cells (Fig. S1B and S1C), consistent with our previous findings using wild-type HIV infection (25). We also quantified HBV DNA by Southern blot in both models of HBV infection and found marked increases in HBV DNA following HIV infection of AD38 cells but not following HIV infection of HBV-infected HepG2-NTCP cells (Fig. S1D and S1E). These results demonstrate a direct impact of HIV infection on the intracellular protein level of HBsAg in hepatocytes, independent of an effect on HBV DNA.

To determine if the increase in HBsAg was a direct effect of HIV infection and not a bystander effect in uninfected cells, we sorted GFP+ and GFP− cells following HIV infection and quantified HBsAg. We detected HIV integration in both GFP+ and GFP− cells but at much lower levels in GFP− cells, as expected (Fig. 2C). Despite detecting HIV integration in both subsets, we only observed an increase in HBsAg in GFP+ cells compared to mock infection (Fig. 2D). These findings suggest that productive infection rather than integration of HIV alone is associated with elevated intracellular HBsAg. Finally, to demonstrate that GFP itself in the pseudotyped HIV virus was not driving increased HBsAg, we infected AD38 cells with another VSV.G-pseudotyped HIV virus, which carries a luciferase reporter instead of GFP and also had deletions in both envelope and vpr HIV genes (VSV.G-NL4-3-ΔEnv-ΔVpr-Luc) for 4 days. Consistent with findings using a GFP-expressing virus, infection with the luciferase virus led to high levels of HIV integration and an upregulation of intracellular HBsAg (Fig. 2E and F). Taken together, our results demonstrate that productive HIV infection is associated with an increase in HBsAg expression in hepatocytes producing or infected by HBV, and this is a result of direct infection and not a bystander effect.

### HIV infection upregulates HBs mRNA level in HBV-expressing hepatocytes

**Fig 3:**
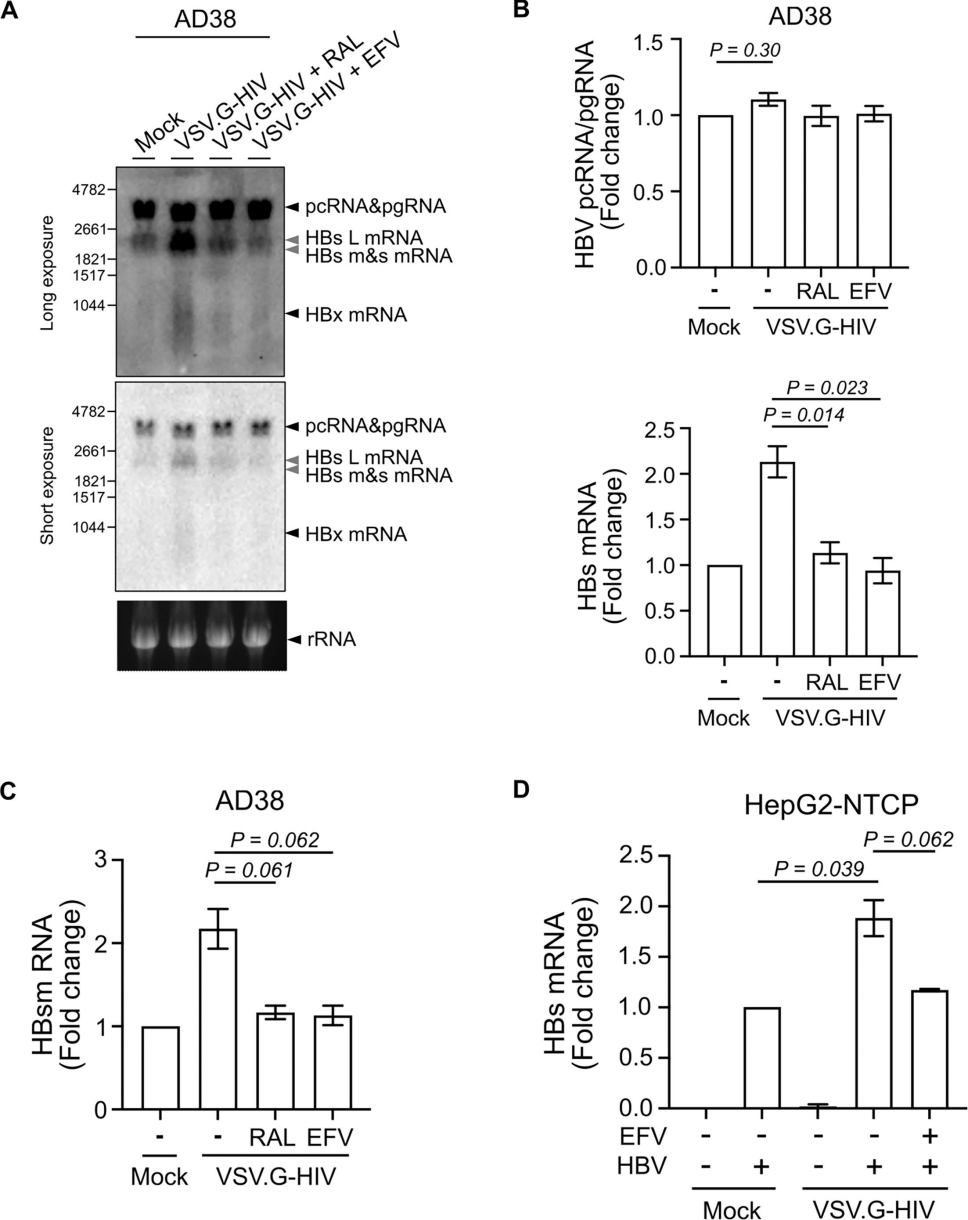
Productive HIV infection leads to a twofold increase in HBs mRNA in AD38 and HepG2-NTCP cells. (**A**) Northern blot of RNA extracted from AD38 cells 5 days post VSV.G-pseudotyped HIV infection. Cells were treated with or without 10 µM RAL or 300 nM EFV 24 h before and immediately after HIV infection. A genomic length HBV-DNA probe or a ribosomal probe was used. A long (upper panel) and short (lower panel) exposure time is shown. Arrows in black indicate pcRNA/pgRNA, HBx mRNA or ribosomal RNA. Arrows in gray indicate large (L), medium (M), and small (S) HBs mRNA; Representative example from three separate experiments. (**B**) Fold change of HBV pcRNA/pgRNA and HBs mRNA on Northern blot (**A**) using image density (*N* = 3). (**C**) Fold change of HBs mRNA expression in AD38 cells 5 days post VSV.G-pseudotyped HIV infection. Cells were treated with or without 10 µM RAL or 300 nM EFV 24 h before and after HIV infection. HBs mRNA was quantified by real-time PCR using two sets of specific primers, which quantified either pcRNA/pgRNA (3.5 kb) only or together with HBs mRNAs (3.5 kb + 2.4 kb + 2.1 kb). HBV PreC (pcRNA/pgRNA) was subtracted from PreC and S (pcRNA/pgRNA with HBs mRNAs) and normalized to the expression of the housekeeping gene RPLP0 (*N* = 3). (**D**) Fold change of HBs mRNA level in HepG2-NTCP cells following HBV and VSV.G-pseudotyped HIV infection. Cells were treated with or without 300 nM EFV 24 h before and after HIV infection. HBs mRNA was quantified by real-time PCR and normalized to the expression of the housekeeping gene RPLP0 (*N* = 3). In all graphs, the columns and error bars represent mean and SEM.

The increase in intracellular HBsAg could be a consequence of increased production or reduced release of HBsAg. We showed in an earlier study that HBsAg in the supernatant was not changed by HIV co-infection, consistent with HIV driving increased HBsAg production, coupled with an increase in intracellular retention (25). We, therefore, next quantified HBV RNA levels by Northern blot in the AD38 cell line following HIV infection. HBV pregenomic RNA (pgRNA) remained unchanged in the presence or absence of VSV.G-HIV-EGFP infection. In contrast, HBV surface (HBs) mRNA was increased by twofold upon HIV infection, and the increase was reduced in the presence of raltegravir or efavirenz (Fig. 3A and B).

Next, we performed real-time PCR to precisely quantify the mRNA levels of HBs in both AD38 cells and HBV-infected HepG2-NTCP with and without infection with VSV.G-HIV-EGFP. Because the genome for HBV pcRNA/pgRNA overlaps with HBs RNA, we used two sets of specific primers, which quantified either pcRNA/pgRNA (3.5 kb) only or together with HBs mRNAs (3.5 kb + 2.4 kb + 2.1 kb) and subtracted HBV PreC (pcRNA/pgRNA) from PreC and S (pcRNA/pgRNA with HBs mRNAs). HBs mRNA levels were upregulated two-fold following HIV infection of either AD38 cells or HBV-infected HepG2-NTCP, and this increase was inhibited by both raltegravir or efavirenz (Fig. 3C and D). However, pcRNA/pgRNA levels did not change significantly in either cell line (Fig. S2A and S2B). These results indicate that the increase in HBsAg protein level was a result of increased transcription of HBs mRNA in hepatocytes following productive HIV infection.

### HIV Tat stimulates HBs transcription via CDK9

**Fig 4:**
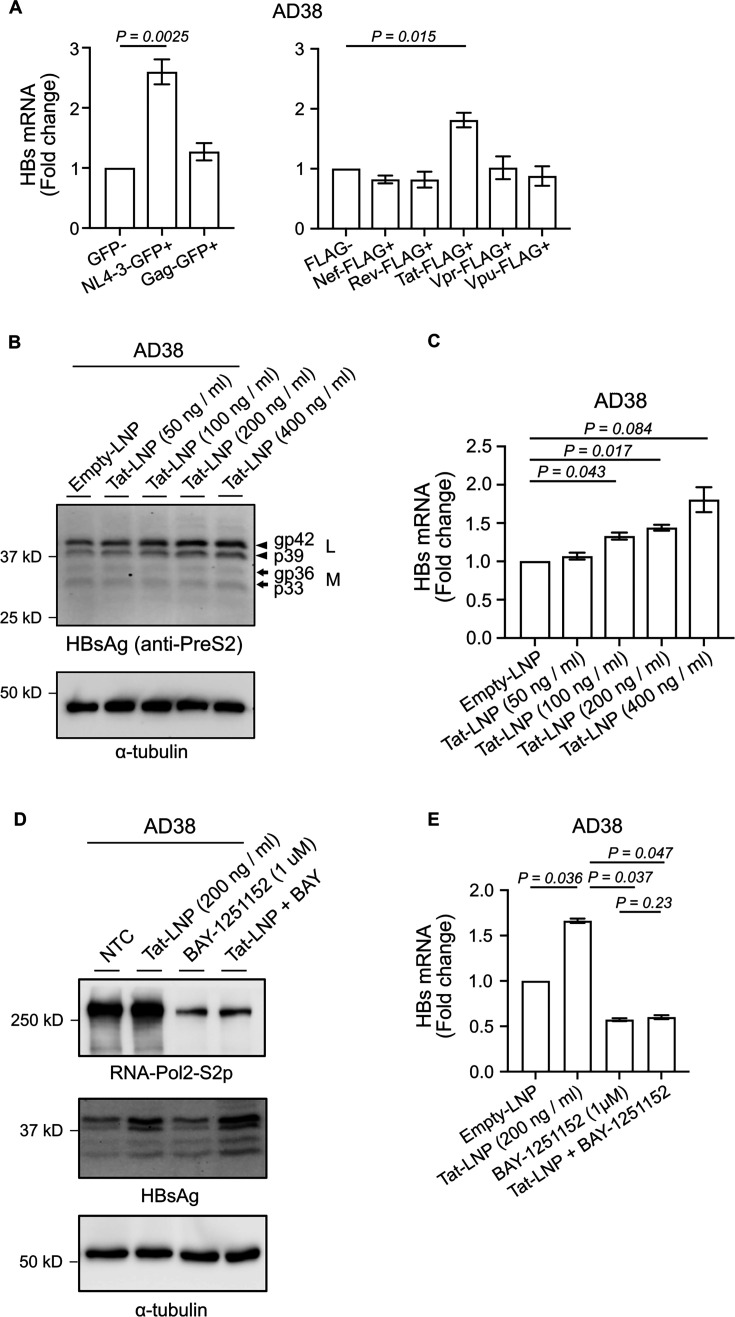
HIV Tat upregulates the transcription level of HBs via CDK9/cyclin T2. (**A**) Fold change of HBs mRNA in AD38 cells following transfection of plasmids expressing either HIV NL4-3-GFP or Gag-GFP (left panel) or FLAG-tagged Nef, Rev, Tat, Vpr, or Vpu (right panel). The cells were transfected with plasmid DNA and enriched following flow sorting, based on expression of either GFP expression or FLAG intracellular staining. HBs mRNA was quantified by real-time PCR and normalized to the expression of the housekeeping gene RPLP0 (*N* = 5). (**B**) Western blot with anti-HBV PreS2 and anti-α-tubulin antibodies using lysates from AD38 cells 2 days post-transfection with lipid nanoparticles (LNPs), which were empty or co-formulated with Tat mRNA (Tat-LNP) ranging from 50 to 400 ng/mL. Results are representative of three experiments. (**C**) Fold change of HBs mRNA level in AD38 cells 2 days post Tat-LNP transfection. HBs mRNA was quantified by real-time PCR and normalized to the expression of the housekeeping gene RPLP0 (*N* = 3). (**D**) Western blot with anti-RNA-Pol2-S2p, anti-HBV PreS2, and anti-α-tubulin antibodies. AD38 cells were transfected with or without 200 ng/mL Tat-LNP for 48 h and treated with or without 1 µM BAY1251152 for another 16 h. Results are representative of two experiments. (**E**) Fold change of HBs mRNA level in AD38 cells transfected with or without 200 ng/mL Tat-LNP for 48 h followed by 1 µM BAY1251152 treatment for another 16 h. HBs mRNA was quantified by real-time PCR and normalized to the expression of the housekeeping gene RPLP0 (*N* = 2). In all graphs, the columns and error bars represent mean and SEM.

To determine whether HIV proteins directly upregulated HBs mRNA level, leading to a higher expression of intracellular HBsAg following productive HIV infection, we transfected AD38 cells with either a plasmid DNA construct expressing the HIV genome with a deletion in envelope (NL4-3-ΔEnv) or a plasmid expressing different HIV proteins with a FLAG or GFP tag. Following a 72-h transfection, the cells were enriched by flow sorting based on GFP expression or intracellular staining of FLAG, and total RNA was then extracted. HBs mRNA was quantified by real-time PCR. Transfection of the NL4-3-ΔEnv plasmid led to an increase in the HBs mRNA level by 2.5-fold (*P* = 0.0025), similar to what we observed with near full-length pseudotyped virus infection. Transfection of the plasmid expressing Tat but not Gag, Nef, Rev, Vpr, or Vpu also led to a twofold increase in HBs mRNA level compared to the relevant negative control (*P* = 0.015; Fig. 4A and S2C). To confirm these findings using a different method, we synthesized the first exon of Tat as mRNA, encapsulated the mRNA into lipid nanoparticles (LNPs), and delivered Tat-LNP to AD38 and TZM-bl cells, as a positive control. TZM-bl cells contain a luciferase reporter under the control of the HIV long terminal repeat (LTR), and following treatment with the Tat-LNP, a dose-dependent increase in luciferase was observed (Fig. S3A). Tat-LNP in AD38 cells also led to a dose-dependent increase in intracellular HBsAg, but with a much lower magnitude than what was demonstrated in the TZM-bl cell line, with a maximum 1.8-fold elevation of HBs mRNA (Fig. 4B, C and S3B), consistent with Tat mediating an increase in HBs transcription.

Given that Tat has been shown to directly activate CDK9 *in vitro* and CDK9 increases HBV transcription and translation (26–28), we tested whether the effects of Tat on HBV replication were mediated by CDK9 by inhibiting P-TEFb/CDK9 with BAY-1251152. We added BAY-1251152 (1 µM) to AD38 cells 48 h following Tat-LNP treatment, and after a further 16 h confirmed activity, as demonstrated by a reduction in phosphorylation of RNA polymerase II (RNA-Pol2-S2p) measured by Western blot (Fig. 4D). Although both pcRNA/pgRNA and HBs mRNA levels were dramatically decreased by the inhibition of CDK9, consistent with inhibition of HBV viral replication, HBs expression remained unchanged with additional Tat-LNP treatment compared to BAY-1251152 alone (Fig. 4E and S3C). These data suggest that the upregulation of HBs transcription by HIV Tat could be rescued by the inhibition of CDK9. However, intracellular protein levels of HBsAg on Western blots were not decreased by this inhibition (Fig. 4D), possibly due to a longer half-life of HBsAg than HBs mRNA within the limited incubation time with BAY-1251152 that we used in these experiments. These results are consistent with a direct upregulation of HBs mRNA by HIV Tat via P-TEFb/CDK9.

## DISCUSSION

In this study, we used a new model of HBV infection of HepG2-NTCP cells with HBV-free virions, followed by single-round infection with a VSV.G-pseudotyped HIV virus to assess how replication of HBV is impacted by co-infection with HIV. We found that productive HIV infection led to a twofold upregulation of HBs mRNA and a marked increase in intracellular production and cellular retention of HBsAg. Overexpression of HIV Tat protein, but not other HIV proteins, by DNA plasmid transfection in the HBV-producing cell line AD38 significantly stimulated HBs mRNA expression. This could be rescued by CDK9 inhibition with BAY-1251152. This study provides new insights into the mechanisms by which HIV directly impacts HBV replication and has implications for understanding adverse liver outcomes in people living with HIV and HBV.

We found a clear increase in both mRNA for HBs and all forms of intracellular HBsAg in HBV-infected hepatocytes following co-infection with HIV (Fig. 2B and 3A), consistent with our previous study using AD38 cells (25). AD38 cells stably express HBV under the control of a tetracycline-responsive cytomegalovirus promoter, while HBV infection of HepG2-NTCP cells more accurately replicates *in vivo* HBV infection (29). The overall HBV infection frequency in HepG2-NTCP cells was approximately 20% by immunofluorescence staining. Given the lower MOI, it is not surprising that the frequency of HBV infection was lower than prior reports (30).

Increased HBV integration could potentially contribute to the upregulation of HBs in HIV co-infected cells. However, we believe this is unlikely as HBV integration is infrequent in chronically infected cell lines (31). Since the budding of both HIV and HBV relies on the endosomal sorting complex required for the transport system (32), HIV co-infection in hepatocytes may also restrict the secretion capacity of HBsAg. Indeed, HBsAg levels in the supernatant were similar in mono and co-infected hepatocytes (Fig. S1B and S1C), consistent with no change in the release of HBsAg from the cytosol. Taken together, this would lead to the accumulation of HBsAg, which can damage infected hepatocytes in chronic hepatitis B (33), via several pathways. First, the intracellular level of L surface protein has been associated with the severity of liver disease caused by coagulative necrosis of hepatocytes in transgenic mice (34). Second, expression of L or S proteins *in vitro* has been shown to induce apoptosis in hepatocyte cell lines (35, 36). Therefore, the accumulation of HBsAg in the presence of HIV-HBV co-infection, even if only in a subset of HIV-infected cells, may well be a driver of adverse liver outcomes in co-infection.

We clearly demonstrated that Tat rather than other HIV viral proteins stimulated HBs mRNA expression. Tat is known to promote HIV transcription by binding to the transactivation response element on the HIV LTR and activating RNA Polymerase II (37, 38). This involves the recruitment of positive transcription elongation factor (P-TEFb) containing CDK9 to the super elongation complex (SEC) (39). The SEC, together with BRD4, can also promote HBV transcription (26), and HBV replication can be inhibited by CDK9 inhibitors (27). We showed that inhibition of CDK9 caused a significant decrease in HBV transcription, including the HBs mRNA (Fig. 4E and S3C). Given that Tat did not increase HBsAg in the presence of CDK9 inhibition, Tat may activate HBs transcription by enhancing the recruitment of P-TEFb to the preS1 and preS2 promoters.

It is possible that Tat could also have an indirect impact on an HBV-infected cell and, therefore, will affect hepatocytes beyond those that are infected with HIV. For example, soluble Tat can be secreted from HIV-infected cells and impact bystander cells (40, 41). Given that soluble Tat can be taken up by neighbouring uninfected cells, and there is only a very small proportion of hepatocytes infected with HIV in the liver (25, 42, 43), Tat could be released from either infected hepatocytes or infected CD4^+^ T cells that migrate through the liver, resulting in a continuous stimulus for HBsAg production. Consistent with this mechanism, soluble Tat can induce apoptosis in neurons in the absence of HIV infection (44). Our findings have implications for some investigative HIV cure strategies such as latency reversal (reviewed in Tanaka et al.) (45), which could potentially be harmful in the setting of HIV-HBV co-infection should there be an increase in Tat protein (46). In addition, the recent identification of Tat inhibitors, although still in pre-clinical development, could play a beneficial role in the management of people with HIV and HBV at high risk of liver disease (47).

This is the first study to show the direct effects of Tat on HBsAg transcription. However, we acknowledge several limitations in our approach. First, we used immortalized cell lines, which have clear differences in gene expression compared to primary hepatocytes (48). However, isolation and adaptation of primary cell culture from liver biopsy can also have a significant unintended impact on the gene expression level (49). Three-dimensional liver organoids derived from adult stem cells have been shown to robustly mimic the functionality of adult hepatocytes (50). Future studies using liver organoids co-cultured with primary T cells could offer further insights into the interactions between HIV and HBV replication. Second, infection with a pseudotyped HIV virus is an artificial model of what happens *in vivo*. Although hepatocytes express very low levels of the HIV receptor (CD4) and co-receptors (CXCR4 or CCR5), we previously showed that, *in vitro*, HIV could infect hepatocytes at very low levels (25). Multiple studies using *in situ* hybridization and, more recently, DNA and RNA scope by our group have clearly identified HIV RNA and DNA in hepatocytes in liver biopsies from patients with HIV off-ART, confirming that HIV can indeed infect hepatocytes *in vivo* (42, 43, 51). The use of a pseudotyped HIV virus allowed for consistent and highly reproducible HIV infection, which greatly facilitated our ability to capture the infrequent but significant impact of HIV infection on HBV replication within a co-infected cell.

In summary, we established an *in vitro* model of HIV-HBV co-infection of hepatocyte cell lines using HBV and VSV.G-pseudotyped HIV viruses and showed a direct and significant impact of HIV co-infection on the HBV life cycle. We showed that the HIV Tat protein increased HBs transcription, production, and intracellular accumulation of HBsAg, which was mediated by CDK9. Inhibiting Tat protein, as currently being explored as part of an HIV cure strategy, could also potentially benefit people with HIV-HBV co-infection. Our findings provide new insights into the direct effects of HIV on HBV replication and have implications for future exploratory treatment strategies for people living with HIV and HBV, specifically those aimed at the cure of either chronic infection.

## MATERIALS AND METHODS

### Cell culture

The human hepatic cell lines HepG2, AD38, and HepG2-NTCP cells were cultured on 0.01% collagen (C9791; Sigma-Aldrich, St. Louis, MO) coated culture dishes in complete medium: minimal essential media (MEM), Dulbecco’s modified Eagle’s medium (DMEM)/F-12 with 400 µg/mL G418 and DMEM with 5 µg/mL puromycin, respectively, supplemented with 10% fetal bovine serum and 2 mM l-glutamine at 37°C in a humidified atmosphere of 5% CO_2_. HEK 293T and TZM-bl cells were cultured in DMEM supplemented with 10% fetal bovine serum and 2 mM l-glutamine at 37°C in a humidified atmosphere of 5% CO_2_.

### HIV pseudotyped virus production and infection

HEK 293T cells in T75 flask were co-transfected with 16 µg NL4-3-ΔEnv with an enhanced GFP (EGFP) gene (a kind gift from Damien Purcell, University of Melbourne, Australia) or NL4-3-ΔEnv-ΔVpr with a luciferase reporter gene (NIH AIDS reagent program) and 4 µg of the envelope plasmid expressing VSV.G using 60 µL FuGENE 6 (E2692; Promega, Madison, WI) according to the manufacturer’s instructions. After overnight incubation, the cells were washed two times and cultured with 12 mL of DMEM complete medium for another 48 h. The supernatant containing pseudotyped HIV was harvested, filtered through a 0.45 µm Filtropur S 0.45 filter (84.1826; Sarstedt, Nümbrecht, Germany) and applied onto 6 mL of 20% sucrose, followed by centrifugation at 26,500 rpm for 1 h in a Sorvall RC-90 ultracentrifuge equipped with an AH-629 rotor. The virus pellet was re-suspended in 1 mL of DMEM complete medium and stored at −80°C until use. Viral stocks were titrated in hepatocytes using the serial dilution method. An MOI of 0.5 was determined by 50% of the hepatocytes expressing EGFP 4 days post-infection. For HIV infection, pseudotyped HIV virus was added directly to the culture medium. The cells were washed two times with phosphate-buffered saline (PBS) 24 h post-infection and incubated for another 72 h until harvest.

### Drug treatment

To inhibit HIV infection, the cells were treated with 10 µM raltegravir or 300 nM efavirenz (NIH AIDS reagent program) 24 h before and immediately after HIV infection. To inhibit CDK9, AD38 cells were treated with 1 µM BAY-1251152 (S8730, Selleckchem, Houston, TX) 48 h after Tat-LNP treatment and incubated for another 16 h until harvest.

### HBV inoculum and infection

Supernatant from AD38 cells containing HBV viral particles was incubated overnight at 4°C with 6% polyethylene glycol (PEG) 8000 (P5431; Sigma-Aldrich), concentrated 100-fold in PBS supplemented with 10% FBS by centrifugation at 11,000 rpm for 1 h at 4°C and stored at −80°C until use. The HBV DNA concentration in VGE per mL was quantified using the COBAS AmpliPrep/COBAS TaqMan HBV test kit v2.0 (Roche Diagnostics, Basel, Switzerland). The concentrated HBV inoculum in DMEM complete medium (600 µL/well of a 12-well plate) supplemented with 4% PEG 8000 was applied onto HepG2-NTCP cells at 800 VGE/cell. The cells were washed two times with PBS 16 h post-infection and incubated for 10 days until harvest, with culture medium change every 2 or 3 days. Secreted HBsAg was quantified as described previously (52).

### RNA extraction, cDNA synthesis, real-time PCR

Cell pellets were homogenized in TRIzol reagent (15596026; Invitrogen, Waltham, MA). Total RNA was extracted and treated with DNase I according to the manufacturer’s instructions. RNA was converted to cDNA using SuperScript III Reverse Transcriptase (18080085; Invitrogen). The quantitative RT-PCR reaction was performed in a Mx3005P QPCR System using Brilliant II SYBR Green QPCR Master Mix (Agilent Technologies, Santa Clara, CA) according to the manufacturer’s instructions. The relative HBs mRNA level was quantified by subtracting HBV PreC (pcRNA and pgRNA) from PreC and S (pcRNA, pgRNA, PreS/L-mRNA, and S-mRNA) after normalization to RPLP0. Primer sequences were, for HBV PreC: forward, 5′-GCCTTAGAGTCTCCTGAGCA-3′; reverse, 5′-GAGGGAGTTCTTCTTCTAGG-3′; for HBV S: forward, 5′-ACCCCTTCTCGTGTTACAGG-3′; reverse, 5′-GAGTGATTGGAGGTTGGGGA-3′; and for RPLP0: forward, 5′-AGATGCAGCAGATCCGCAT-3′; reverse, 5′-GGATGGCCTTGCGCA-3′.

### DNA constructs and transfection

The HIV Tat expressing construct pcDNA3.1-Tat101^AD8^-FLAG was a kind gift from Professor Damien Purcell, University of Melbourne, Australia (53). pPA-GFP-Gag, pCMV6-Nef-FLAG, pCMV6-Vpr-FLAG, and pCMV6-Vpu-FLAG were obtained from the NIH AIDS reagent program. HIV Rev sequence was generated by flanking PCR product of Rev cDNA (forward primer: 5′-CGGAATTCCACC ATGGCAGGAAGAAGCGGAG-3′, reverse primer: 5′-CGACGCGT TTCTTTAGTTCCTGACTCCAATA-3′) and cloned into pCMV6-FLAG vector using EcoRI and MluI sites. Plasmid transfection to AD38 cells was performed using FuGENE 6 according to the manufacturer’s instructions. The cells were harvested 72 h post-transfection.

### mRNA synthesis and LNPs

The coding region of NL4-3-ΔEnv-EGFP was amplified, and a T7 promoter sequence (5′-GAAATTAATACGACTCACTATAGG-3′) and a 37 nt poly-A sequence were added through overhang PCR using Platinum Taq High Fidelity polymerase (11304011; Invitrogen). Resulting reaction products were cleaned up using a QIAquick PCR purification kit (28104; Qiagen, Hilden, Germany) as per the manufacturer’s instructions. *In vitro* transcription was performed using the mMESSAGE mMACHINE T7 Transcription Kit (AM1344; Invitrogen) as per the manufacturer’s instructions, which includes a GG Cap0 for co-transcriptional mRNA capping. 400–600 ng of PCR product was loaded into each 20 µL reaction. Reactions were left for 4 h to maximize mRNA yield. Quality control of the synthesized mRNA was performed in two ways: mRNA size, integrity, and purity were assessed through gel electrophoresis using a TapeStation instrument (Agilent Technologies), after which the concentration was determined through fluorescence detection using the Quant-iT RiboGreen RNA Assay Kit (R11490, Invitrogen) as per the manufacturer’s low-range protocol.

LNPs encapsulating HIV Tat mRNA were synthesized through microfluidic mixing using a NanoAssemblr Spark (Precision Nanosystems, Vancouver, Canada). Briefly, ionizable lipids, D-Lin-MC3-DMA (MC3) (S6683, Selleckchem), 1,2-distearoyl-sn-glycero-3-phosphocholine (DSPC) (850365P, Avanti Polar Lipids, Alabaster, AL), cholesterol (C8667, Sigma-Aldrich) and 1,2-Dimyristoyl-rac-glycero-3-methoxypolyethylene glycol-2000 (DMG-PEG2000) (880151P, Avanti Polar Lipids) were resuspended in 100% ethanol at 10 mM and stored at −30°C until further use. Lipids were mixed at a molar ratio of 50:10:38.5:1.5 of MC3:DSPC:cholesterol:DMG-PEG2000. Immediately prior to microfluidic mixing, RNA constructs were diluted to 150 ng/µL in 30 mM RNase-free sodium acetate buffer, pH 4.0. Aqueous and organic phases were then mixed at a flow rate ratio of 1.8 (aqueous):1 (organic) (N/P ratio of 6:1), and resulting LNPs were diluted fourfold into PBS to neutralise the pH. LNP size and polydispersity were determined using dynamic light scattering (Zetasizer Ultra, Malvern Panalytical, Malvern, UK). RNA encapsulation efficiency and total RNA concentration of the LNP solution were determined using a modified RiboGreen assay. Briefly, RNA concentration measurements were performed on LNP samples as per the manufacturer’s low-range assay protocol after 30-min incubation in either standard TE buffer (for free RNA measurement) or in TE buffer with 1% Triton X-100 to release encapsulated RNA (for total RNA measurement). LNPs were either stored at 4°C for a maximum of 2 weeks or frozen at −80°C in the presence of 20% wt/vol sucrose for cryoprotection.

### Quantification of HIV integration

Cells were lysed in 10 mg/mL proteinase K buffer. HIV integrated DNA was quantified as described previously and normalized to the CCR5 gene as a surrogate for the number of cells per reaction (54–56).

### Antibodies

Commercial primary antibodies used for Western blotting, immunofluorescence and flow cytometry were rabbit anti-HBV preS2 (PA5-22820; Invitrogen), rabbit anti-HBsAg (NB100-62652; Novas Biologicals, Littleton, CO), mouse anti-FLAG M2 (F1804; Sigma-Aldrich), rabbit anti-GFP (ab290; Abcam, Cambridge, UK), rabbit anti-GAPDH (2118; Cell Signaling Technology, Danvers, MA), rabbit anti-RNA polymerase II CTD, phospho S2 (ab193467; Abcam), and SureLight APC mouse anti-DDDDK tag (ab72569; Abcam). Anti-mouse IgG-HRP and anti-rabbit IgG-HRP or Alexa Fluor 488 and 568 (A11001, A11008, A11011, and A11031; Invitrogen) were used as secondary antibodies for Western blotting or immunofluorescence.

### Immunofluorescence microscopy

HepG2-NTCP cells grown on collagen-coated coverslips were washed with PBS, fixed with 4% paraformaldehyde (PFA) for 15 min, permeabilized with 0.5% Triton X-100 for 5 min and blocked for 1 h in 5% bovine serum albumin (BSA; A7906; Sigma-Aldrich) in PBS at room temperature. Coverslips were incubated with primary antibodies diluted in blocking buffer at 4°C overnight, followed by three washes with 0.05% Tween-20 in PBS. The cells were then incubated with secondary antibodies conjugated with Alexa Fluor 488 or 568 for 1 h at room temperature. After three washes with 0.05% Tween-20 in PBS, DNA was counterstained with DAPI (D1306; Invitrogen). Coverslips were mounted with FluorSave reagent (345789; Merck, Kenilworth, NJ). Images were obtained using a confocal fluorescence microscope LSM700 (Carl Zeiss, Jena, Germany) equipped with a 63× Plan Apochromat oil immersion objective (NA 1.4) and built-in laser scanning unit, at RT. Images were acquired and analyzed using ZEN (Carl Zeiss).

### Analysis of HBV protein, RNA, and DNA

For Western blotting, the cells were harvested, washed once with PBS and lysed in radioimmunoprecipitation assay buffer (50 mM Tris-HCl pH7.4, 150 nM NaCl, 1% NP-40, 0.1% SDS, 50 mM NaF, and 2 mM EDTA) containing 1× Halt protease inhibitor Cocktail (1862209, Thermo Fisher Scientific, Waltham, MA). After centrifugation at 10,000 × *g* for 10 min at 4°C, the supernatant was mixed with 2× Laemmli sample buffer containing 200 mM dithiothreitol and boiled at 95°C for 3 min. For Northern blotting, 30 µg of RNA from each sample was electrophoresed through 1% agarose glyoxal gels before transfer to nylon membranes using the NorthernMax-Gly Kit (AM194; Invitrogen) according to the manufacturer’s instructions. Membrane was probed using a genomic length HBV-DNA probe as previously described (57). For Southern blotting, a DIG-labeled 2.5 kb DNA probe (forward, 5′-AAGGTGGGAAACTTTACTGGGC-3′; reverse, 5′-GGCAAAAACGAGAGTAACTC-3′) was used as previously described (Roche Diagnostics) (52).

### Flow cytometry

The cells were dispersed to single-cell suspension using Trypsin-EDTA, passed through a 40 µm strainer (Sarstedt, Nümbrecht, Germany), washed once with PBS and incubated with Fixable Viability Stain 450 (562247; BD Biosciences, Franklin Lakes, NJ) for 20 min at room temperature. The cells were washed again with PBS and fixed in PBS containing 1% formaldehyde. All data were acquired on a LSRFortessa Cell Analyzer (BD Biosciences) and were analyzed using FlowJo 9.9.6. Live and single cells were gated using forward and side scatter plots. For sorting, the cells were re-suspended in PBS containing 10% BSA and 10 mM EDTA instead of 1% formaldehyde and sorted on a FACSAria III Cell Sorter (BD Biosciences) based on GFP expression. Isolation of high-quality RNA following intracellular sorting was performed using the method for analysing RNA following intracellular sorting (MARIS)(58). The cells were fixed and permeabilized in 4% PFA supplemented with 100 U/mL RNAse OUT (1077019; Invitrogen) for 30 min on ice. The cells were pelleted by centrifugation at 500 × *g* for 3 min at 4°C, washed in wash buffer: PBS containing 0.2% BSA and RNAse OUT and incubated with SureLight APC anti-FLAG antibody M2 (ab72569; Abcam) diluted in PBS with 1% BSA and RNAse OUT for 1 h at 4°C. The cells were washed two times, re-suspended in PBS with 0.5% BSA and RNAse OUT and sorted on a BD FACSAria III Cell Sorter based on the expression of FLAG.

### Statistical analysis

All data shown represent the mean value of at least three independent experiments. Error bars represent the standard error of the mean. *P* values for bar plots were obtained by paired *t*-test and represent a comparison of all cells analyzed in the indicated cell populations. For all multiple comparisons, one-way ANOVA with Geisser-Greenhouse correction was performed. A *P* value of less than 0.05 was considered significant. All analysis was performed using GraphPad Prism 9 (GraphPad, La Jolla, CA).

## SUPPLEMENTAL MATERIAL

Figure S1 spectrum.00809-25-s0001.tiff Pseudotyped HIV infection of HBV-expressing hepatocytes leads to efficient HIV integration and production.

Figure S2 spectrum.00809-25-s0002.tiff HIV infection up-regulates HBs mRNA level in HBV-expressing hepatocytes.

Figure S3 spectrum.00809-25-s0003.tiff HIV Tat stimulates HBs transcription via CDK9.

Supplemental legends spectrum.00809-25-s0004.docx Legends for Fig. S1 to S3.

ASM does not own the copyrights to Supplemental Material that may be linked to, or accessed through, an article. The authors have granted ASM a non-exclusive, world-wide license to publish the Supplemental Material files. Please contact the corresponding author directly for reuse.
