# Supplementary figures and images for: Hepatitis B surface antigen is upregulated by HIV Tat in an HIV–hepatitis B virus co-infection model system
Source: Microbiol Spectr. 2025 Jul 23;13(9):e00809-25. doi: 10.1128/spectrum.00809-25 (PMC12403812; doi:10.1128/spectrum.00809-25)

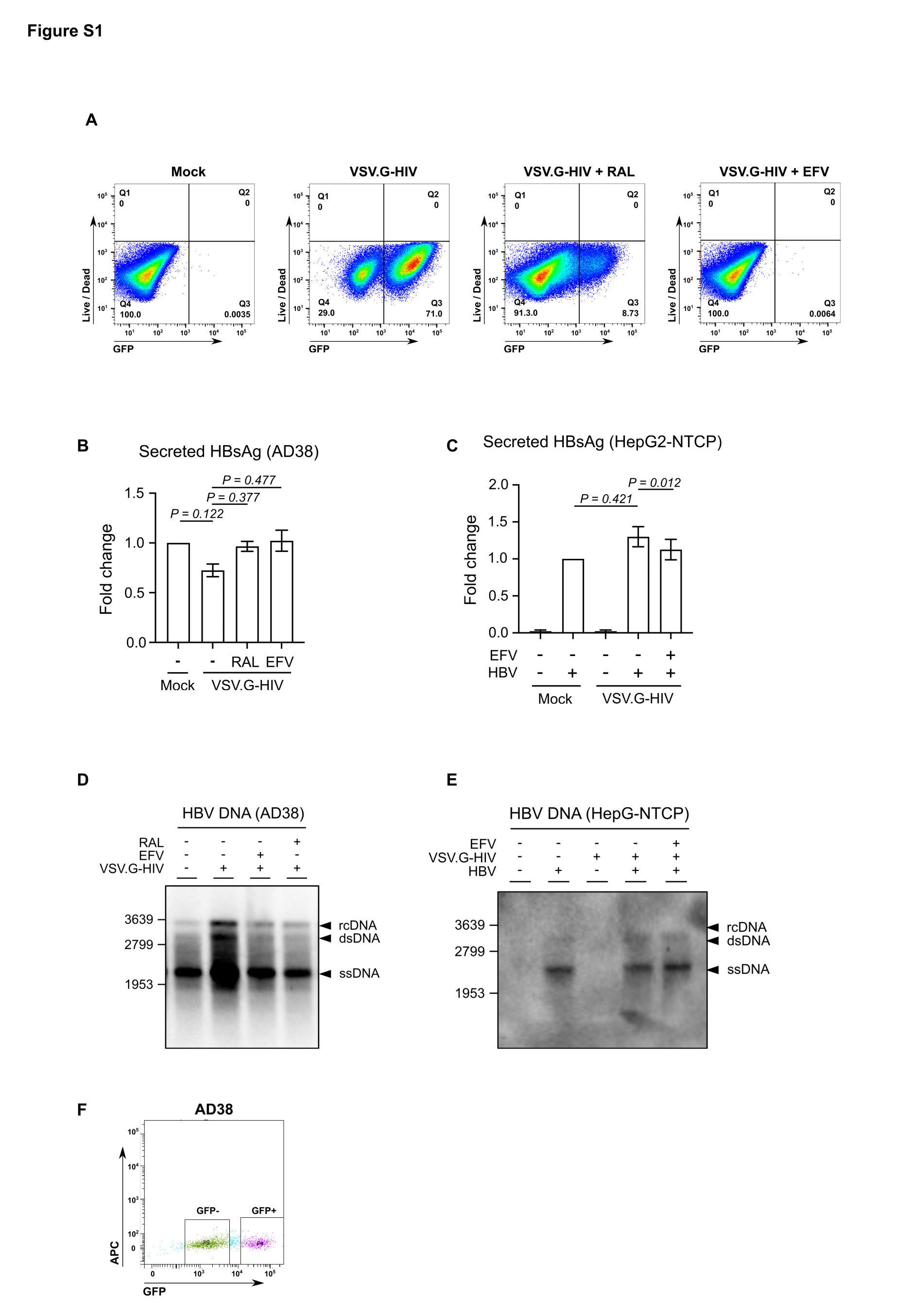

Supplement: Figure S1 — Pseudotyped HIV infection of HBV-expressing hepatocytes leads to efficient HIV integration and production. [file spectrum.00809-25-s0001.tiff]

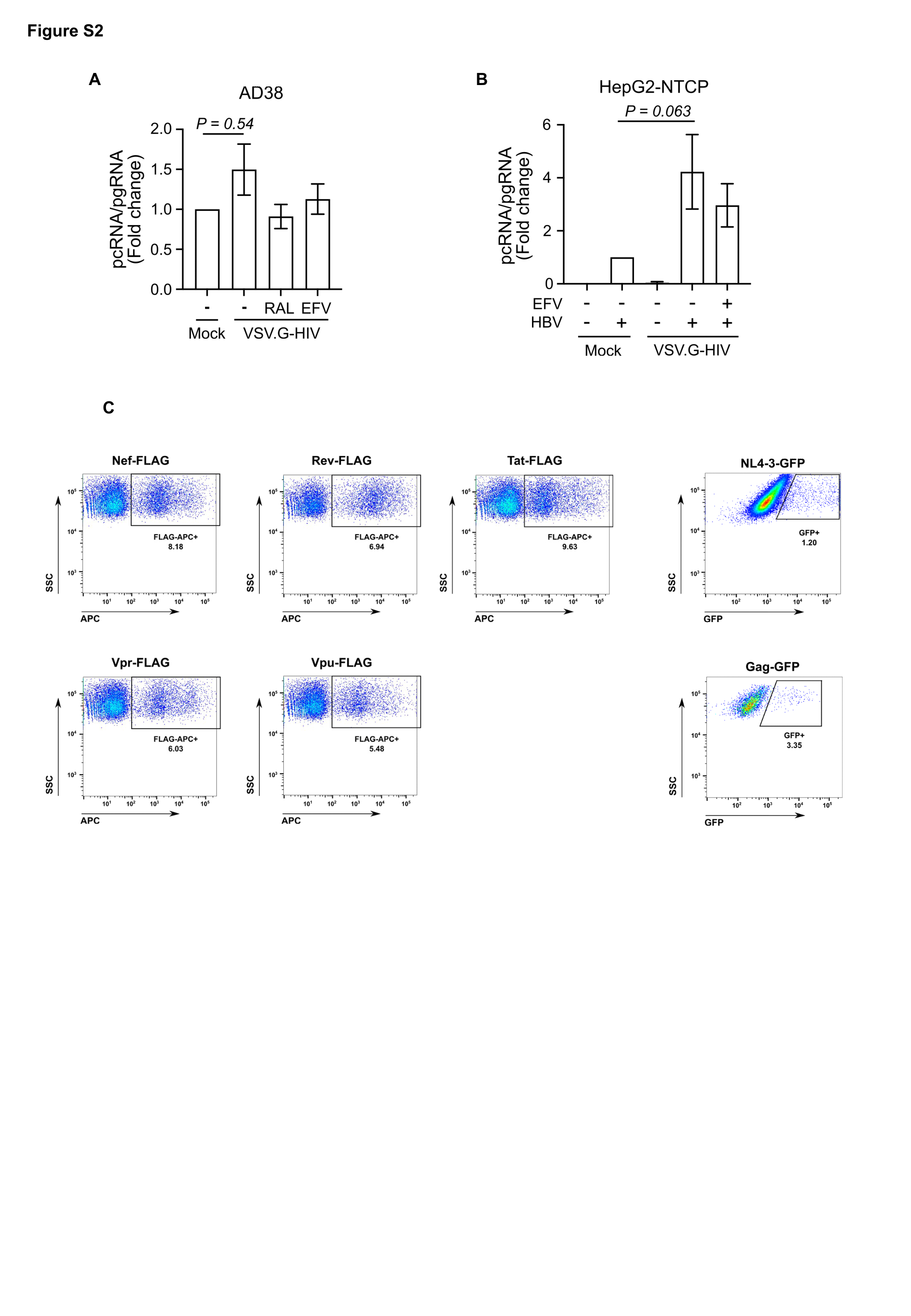

Supplement: Figure S2 — HIV infection up-regulates HBs mRNA level in HBV-expressing hepatocytes. [file spectrum.00809-25-s0002.tiff]

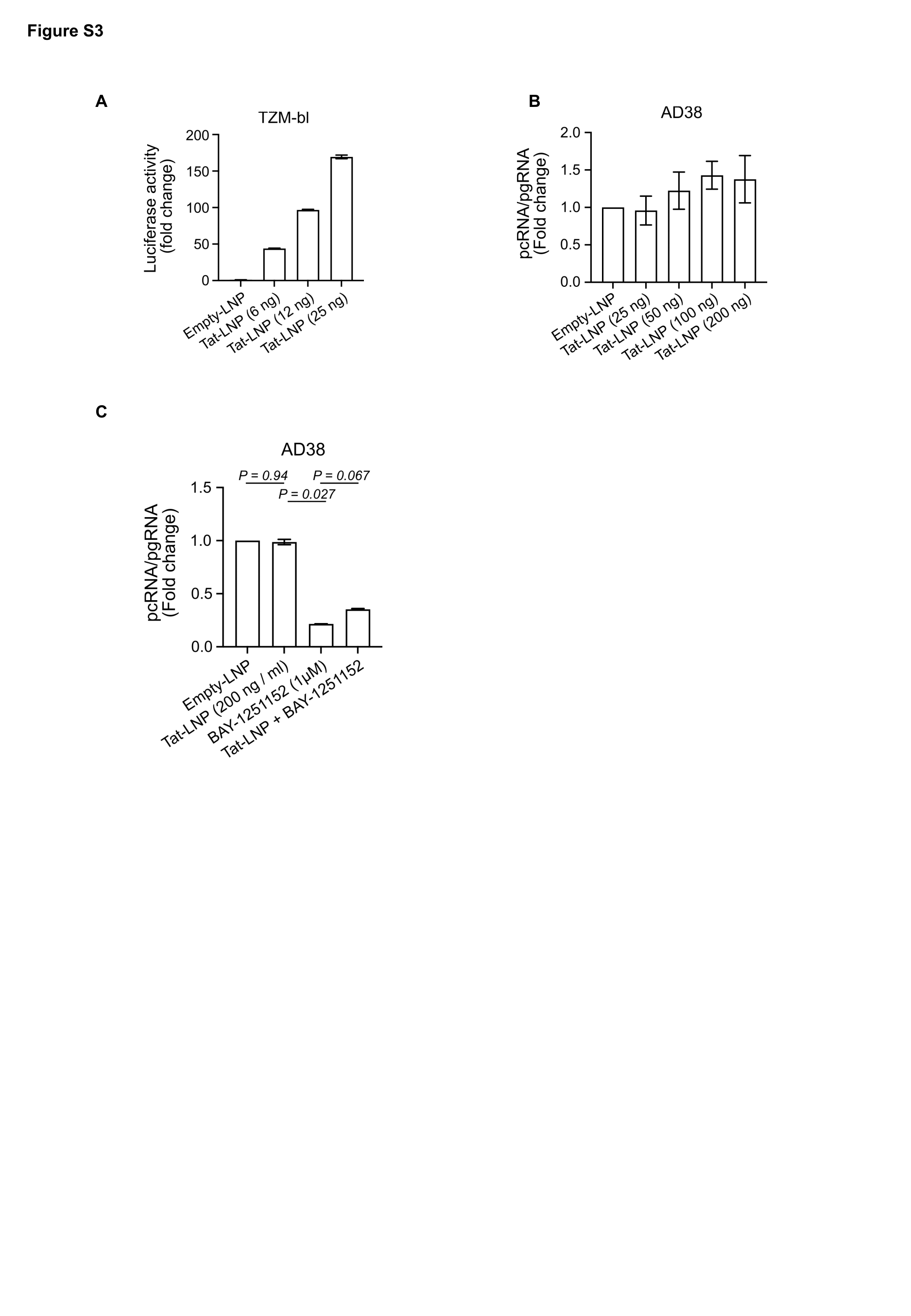

Supplement: Figure S3 — HIV Tat stimulates HBs transcription via CDK9. [file spectrum.00809-25-s0003.tiff]
