# Supplementary material for: Hepatitis B surface antigen is upregulated by HIV Tat in an HIV–hepatitis B virus co-infection model system
Source: Microbiol Spectr. 2025 Jul 23;13(9):e00809-25. doi: 10.1128/spectrum.00809-25 (PMC12403812; doi:10.1128/spectrum.00809-25)
Supplement: Supplemental legends — Legends for Fig. S1 to S3. [file spectrum.00809-25-s0004.docx]

**Supplemental figure legends**

**Figure S1** (A) Quantification of GFP+ AD38 cells by flow cytometry 5-day post VSV.G-pseudotyped HIV infection. Cells were stained with Fixable Viability Stain 450 and gating was made on the basis of forward and side scatter (FSC and SSC, respectively) and expression of green fluorescent protein (GFP). Cells were treated with or without 10 μM RAL or 300 nM EFV 24 h before and immediately after HIV infection. Results are representative of at least three experiments. (B) Fold change increase of secreted HBsAg in AD38 cells 5-day post VSV.G-pseudotyped HIV infection. Cells were treated with or without 10 μM RAL or 300 nM EFV 24 h before and immediately after HIV infection; *N* = 3. (C) Fold change increase of secreted HBsAg in HepG2-NTCP cells with HBV and VSV.G-pseudotyped HIV infection. Cells were treated with or without 300 nM EFV 24 h before and immediately after HIV infection; *N* = 3. (D) HBV DNA level in AD38 cells 5-day post-VSV.G-pseudotyped HIV infection. Cells were treated with or without 10 μM RAL or 300 nM EFV 24 h before and immediately after HIV infection. Results are representative of three experiments. (E) HBV DNA level in HepG2-NTCP cells infected with HBV and VSV.G-pseudotyped HIV infection. Cells were treated with or without 300 nM EFV 24 h before and immediately after HIV infection. HBV DNA was detected by Southern blot with a genomic length HBV-DNA probe. rcDNA, relaxed circular DNA; dsDNA, double-stranded DNA; ssDNA, single-stranded DNA. Results are representative of at least three experiments. In all graphs, columns + error bars represent mean and SEM.

**Figure S2** (A) Fold change expression of HBV pcRNA/pgRNA in AD38 cells 5-day post VSV.G-pseudotyped HIV infection. Cells were treated with or without 10 μM RAL or 300 nM EFV 24 h before and immediately after HIV infection. pcRNA/pgRNA level was quantified by real time PCR and normalized to expression of the house keeping gene RPLP0; *N* = 3. (B) Fold change expression of HBV pcRNA/pgRNA levels in HepG2-NTCP cells following infection with HBV and VSV.G-pseudotyped HIV infection. Cells were treated with or without 300 nM EFV 24 h before and after HIV infection. pcRNA/pgRNA level was quantified by real-time PCR and normalized to expression of the housekeeping gene RPLP0; *N* = 3. (C) Flow cytometry plots of AD38 cells that expressed either GFP or FLAG tag after plasmid DNA transfection. Cells expressing FLAG-tagged protein were intracellularly stained with anti-FLAG antibody and anti-mouse antibodies bound to Allophycocyanin (APC). Results are representative of five experiments. In all graphs, columns + error bars represent mean and SEM.

**Figure S3** (A) Fold change expression of luciferase activity in TZM-bl cells following transfection with lipid nanoparticles (LNP) that were either empty or co-formulated with mRNA Tat (Tat-LNP). Cells were transfected with 25 ng empty-LNP or Tat-LNP from 6 ng to 25 ng per well in a 96-well plate. LTR luciferase activity was assessed 2-day post transfection. *N* = 2 (B) Fold change of HBV pcRNA/pgRNA level in AD38 cells 2-days post Tat-LNP transfection. Cells were treated with empty LNP, or Tat-LNP from 25 ng to 200 ng per well in a 24-well plate. pcRNA/pgRNA was quantified by real time PCR and normalised to the expression of the house-keeping gene RPLP0; *N* = 3. (C) Fold change of HBV pcRNA/pgRNA in AD38 cells transfected with or without 200 ng / ml Tat-LNP for 48 h followed by 1 µM BAY1251152 treatment for another 16 h. pcRNA/pgRNA was quantified by real-time PCR and normalized to the expression of the housekeeping gene RPLP0; *N* = 3. In all graphs, columns + error bars represent mean and SEM.
